# From Intestinal Permeability to Dysmotility: The Biobreeding Rat as a Model for Functional Gastrointestinal Disorders

**DOI:** 10.1371/journal.pone.0111132

**Published:** 2014-10-29

**Authors:** Tim Vanuytsel, Christophe Vanormelingen, Hanne Vanheel, Tatsuhiro Masaoka, Shadea Salim Rasoel, Joran Tóth, Els Houben, Kristin Verbeke, Gert De Hertogh, Pieter Vanden Berghe, Jan Tack, Ricard Farré

**Affiliations:** 1 Translational Research Center for Gastrointestinal Disorders (TARGID), University of Leuven, Leuven, Belgium; 2 Department of Pathology, University Hospital, University of Leuven, Leuven, Belgium; 3 Centro de Investigación Biomédica en Red de Enfermedades Hepáticas y Digestivas (CIBERehd), Instituto de Salud Carlos II, Barcelona, Spain; University of California, Los Angeles, United States of America

## Abstract

**Background:**

Impaired intestinal barrier function, low-grade inflammation and altered neuronal control are reported in functional gastrointestinal disorders. However, the sequence of and causal relation between these events is unclear, necessitating a spontaneous animal model. The aim of this study was to describe the natural history of intestinal permeability, mucosal and neuromuscular inflammation and nitrergic motor neuron function during the lifetime of the BioBreeding (BB) rat.

**Methods:**

Normoglycemic BB-diabetes prone (DP) and control rats were sacrificed at different ages and jejunum was harvested to characterize intestinal permeability, inflammation and neuromuscular function.

**Results:**

Both structural and functional evidence of increased intestinal permeability was found in young BB-DP rats from the age of 50 days. In older animals, starting in the mucosa from 70 days and in half of the animals also in the muscularis propria from 110 days, an inflammatory reaction, characterized by an influx of polymorphonuclear cells and higher myeloperoxidase activity, was observed. Finally, in animals older than 110 days, coinciding with a myenteric ganglionitis, a loss of nitrergic neurons and motor function was demonstrated.

**Conclusion:**

In the BB-rat, mucosal inflammatory cell infiltration is preceded by intestinal barrier dysfunction and followed by myenteric ganglionitis and loss of nitrergic function. This sequence supports a primary role for impaired barrier function and provides an insightful model for the pathogenesis of functional gastrointestinal disorders.

## Introduction

Functional gastrointestinal disorders (FGID) like irritable bowel syndrome (IBS) and functional dyspepsia are characterized by bothersome gastrointestinal complaints in the absence of an underlying organic cause that readily explains the symptoms [1], [2]. Despite the high prevalence, the pathophysiology of FGID remains incompletely understood and the current treatment options are limited and have suboptimal efficacy. Impaired intestinal barrier function [3]–[5], low-grade immune activation [4]–[6], and altered neuronal control of gastrointestinal motility [7], [8], have been suggested to be involved in the pathogenesis. An attractive and often-cited disease model for FGID, and also for chronic inflammatory bowel disease (IBD), is based on luminal antigen penetration through an impaired intestinal barrier leading to immune activation in the intestinal wall [9]. On the other hand, it is well recognized that increased intestinal permeability may also be a consequence of inflammatory changes [10]–[12]. The current data on intestinal permeability in human intestinal disorders are mainly associative, with a possible exception of Crohn’s disease [13], [14], and celiac disease [15]. Especially in FGID, a cause-consequence relationship between the observed alterations in permeability, immune activation and motility disturbances, has not been established so far and is subject of ongoing debate [11]. The distinction is relevant since therapeutic interventions aimed at restoring barrier function could represent a novel treatment approach to several gastrointestinal disorders. A spontaneous animal model sharing key intestinal characteristics of human FGID would be instrumental to separate cause from consequence and to study future treatments.

The BioBreeding (BB) rat is a well-established animal model for type 1 diabetes. The BB-rat consists of two strains, the diabetes-resistant (BB-DR) and the diabetes-prone (BB-DP) strain. Hyperglycemia develops in 50–95% of the BB-DP animals depending on the substrain, diet and housing conditions [16], [17]. The hyperglycemic BB-DP rat displays increased intestinal permeability prior to the development of diabetes [16], [18], [19], mucosal [20], [21] and neuromuscular [22] inflammation and loss of nitrergic motor neuron function [17], [22]. We have previously reported that the development of the inflammatory enteropathy and loss of nitrergic neurons in the BB-rat also occur in BB-DP animals which do not develop diabetes [23]. These features potentially identify the normoglycemic BB-DP rat as a suitable animal model for inflammatory neuromuscular dysfunction. However, current data on intestinal permeability and functional neuromuscular data are limited to diabetic animals, in which diabetes potentially is a confounding factor. Moreover, it is unclear how permeability, inflammation and intestinal nitrergic neuropathy evolve over time and how they temporally relate to each other.

The goal of the current study was to describe in detail the evolution over time of intestinal barrier function, mucosal and neuromuscular inflammation and loss of nitrergic motor neuron function in the normoglycemic BB-DP rat. We hypothesize that intestinal permeability is an early feature of the normoglycemic BB-DP rat, preceding inflammation and neuromuscular alterations, suggesting its disease-initiating role.

## Materials and Methods

### Animals

Breeding pairs of BB-DP and BB-DR were obtained from the Animal Resources Division of Health Canada, Ottawa, ON, Canada and further bred in the conventional animal facility of the University of Leuven. For the current study, we included male BB-DR and normoglycemic BB-DP rats to avoid the contribution of hyperglycemia to the observed differences in permeability, inflammation and neuromuscular function. Rats were housed in wire-meshed cages and had *ad libitum* access to drinking water and standard rat chow. Glycemia was determined on tail blood at least every 7 days with a glucocard-memory-2 device (Menarini, Zaventem, Belgium) in both BB-DR and BB-DP rats, starting from 70 days of age. This time point was chosen because no hyperglycemia was detected in our colony before this age in previous studies. Onset of diabetes was confirmed by glycemia >250 mg/dL on two occasions, which led to exclusion from the study cohort. In our colony, around 50% of BB-DP rats evolve to hyperglycemia with an onset between 85 and 155 days. None of the BB-DR rats developed hyperglycemia. In the remainder of the text, BB-DR rats will be referred to as “control rats” and normoglycemic BB-DP rats as ‘BB-DP rats’. All animal experiments were approved by the ethics committee for animal experiments of the University of Leuven.

### Experimental design

Control and BB-DP rats were sacrificed by cervical dislocation and exsanguination at different ages: 30, 50, 70, 90, 110, 160 and 220 days. These time points were selected based on previously published data indicating increased intestinal permeability in hyperglycemic BB-DP between 50 and 70 days, prior to the onset of diabetes [18], [19]. We previously reported loss of nitrergic neurons and altered contractility in hyperglycemic animals at 220 days [17]. The other time points were added to allow a more detailed time course of the events and to determine whether the permeability defect was already present shortly after weaning age. At each time point at least 7 rats per group were studied. The jejunum, defined as the first half of the small intestine, excluding the first 5 cm after the stomach, was dissected and stored in ice-cold Krebs buffer (120.9 mmol L^−1^ NaCl, 5.9 mmol L^−1^ KCl, 1.2 mmol L^−1 ^MgCl_2_, 1.2 mmol L^−1^ NaH_2_PO_4_, 14.4 mmol L^−1^ NaHCO_3_, 2.5 mmol L^−1^ CaCl_2_ and 11.5 mmol L^−1^ glucose; all purchased from Merck, Overijse, Belgium), continuously gassed with carbogen (95%O_2_, 5%CO_2_) until further processing.

### Permeability assays

#### *In vitro* intestinal permeability

Three segments of mid-jejunum of each animal were mounted in modified Ussing chambers (Mussler Scientific Instruments, Aachen, Germany) without removal of the seromuscular layer, with an exposed area of 0.096 cm^2^. The mucosal and serosal compartments were filled with Krebs-Ringer bicarbonate buffer, supplemented with 10 mM mannitol and 10 mM glucose respectively. Solutions were kept at 37°C and gassed with carbogen. Transmucosal potential difference was continuously monitored using Ag/AgCl electrodes. The transepithelial electrical resistance (TEER) was calculated according to Ohm’s law from the voltage deflections induced by bipolar current pulses of 50 µA every 60 s with a duration of 200 ms. The TEER values were registered for each tissue at 30 min intervals. The average TEER between 90 and 120 minutes was calculated over the three tissues per animal. These time points were selected because a stable TEER plateau is reached in most of the tissues between 90 and 120 minutes after mounting.

Macromolecular flux was studied by adding 20 kDa dextrans conjugated to fluorescein isothiocyanate (FITC-Dx20, final concentration 1 mg/mL, Sigma-Aldrich, Diegem, Belgium) to the mucosal compartment after a 40 minute stabilization period in tissues of animals of 30, 50, 70, 90 and 110 days. Fluorescence was determined in samples from the serosal side, taken with 30 min intervals during 90 minutes. The fluorescence values were converted to pmol/cm^2^ based on a standard curve made for each analysis. Average cumulative passage of FITC-Dx20 90 minutes after adding the probe was calculated for each animal.

#### *In vivo* small intestinal permeability

*In vivo* permeability was quantified by a differential urinary sugar excretion test in rats of 50, 70 and 90 days (at least 7 per group per time point) as previously described [18]. Briefly, a 2 mL solution containing 120 mg of lactulose and 80 mg of mannitol was administered by oral gavage after a 6 hour fast. Urine was collected in metabolic cages during 24 hours in recipients containing 100 µL of a 10 mg/mL chlorhexidine solution to prevent bacterial degradation. Urine was filtered through a 450 nm syringe filter (Millipore, Overijse, Belgium) immediately after collection and stored at −20°C until further processing. Urinary concentrations of lactulose and mannitol were determined by high performance liquid chromatography (HPLC). The internal standard cellobiose (100 µL, 80 mg/dL) was added to the urine sample (10–400 µL) and to a standard solution (20–100 µL) containing mannitol (40 mg/dL or 400 mg/dL) and lactulose (8 mg/dL or 80 mg/dL). All samples were diluted to 500 µL with demineralized water. Twenty µL of the diluted samples was analyzed by HPLC (Alliance 2695, Waters, Zellik, Belgium) which was equipped with a Prevail Carbohydrate column (250 mm×4.6 mm i.d., 5 µm particle size; Grace, Deerfield, MA, USA). The chromatographic separation was carried out isocratically with 75% acetonitrile/25% MilliQ water for 16 minutes. The effluent was analyzed by an evaporative light scattering detector (ELSD 3300, Grace, Deerfield, MA, USA) with a N_2_ flow of 1.5 L/min at 40°C and with a gain of 16. Data were processed using Empower 2.0 (Waters, Zellik, Belgium). The limits of detection were 1.0 and 1.2 mg/L for lactulose and mannitol respectively. The urinary lactulose to mannitol ratio (LMR) served as a measure for intestinal permeability [18], [24]. Mannitol is a small sugar alcohol, which crosses the intestinal epithelial barrier via the paracellular pathway in villus and crypt regions. In this way, urinary excretion of mannitol serves as a measure for the surface of the small intestinal epithelium [24]. In contrast, lactulose, a disaccharide which is double in size, permeates only to a very limited extent in normal conditions and in the crypt regions only. In case of impaired intestinal barrier function, the passage of lactulose will increase contrary to the unaltered excretion of mannitol, resulting in a higher LMR [24].

### Myeloperoxidase activity

The myeloperoxidase (MPO) activity, abundantly expressed in neutrophils and to a lesser extent also in monocytes/macrophages, was determined by a spectrophotometric assay, based on the conversion of O-dianisidine to its colored oxidized form in the presence of H_2_O_2_ [25]. Briefly, the mucosa and submucosa were separated from the muscularis propria by gentle dissection in NaCl 0.9% and immediately snap-frozen in liquid N_2_. Extraction of MPO was performed by homogenization of the tissue in a 0.5% hexadecyltrimethylammonium bromide (Sigma-Aldrich, Diegem, Belgium) buffer followed by two freeze-thaw cycles and a centrifugation step. The absorbance of the supernatant was continuously measured over 20 minutes at 460 nm after addition of H_2_O_2_ (2.13×10^−4^ M) and O-dianisidine hydrochloride (5.23×10^−4^ M) (Sigma-Aldrich). One unit of MPO is defined as the enzyme activity converting 1 µmol of H_2_O_2_ per minute at 25°C. MPO activity was measured in the mucosa and the muscularis propria. Results were expressed in U/mg tissue.

### Real-time PCR

After separation of the mucosa and submucosa from the underlying layers by gentle dissection, the tissue was homogenized in TRIzol reagent (Invitrogen, Ghent, Belgium) and stored at −80°C until further processing. Total RNA was extracted and further purified using a High Pure RNA isolation kit (Roche Diagnostics, Vilvoorde, Belgium) according to the manufacturer’s instructions. c-DNA was synthesized from 2 µg RNA using the qScript cDNA Supermix kit (Quanta Biosciences, Gaithersburg, MD, USA). The real-time PCR reaction was performed on a LightCycler 480 system with SYBR Green I Master mix (Roche Diagnostics, Vilvoorde, Belgium). Specific primers for permeability- and inflammation-related genes were designed using NCBI Primer-Blast (Table S1). A three-step amplification program was used: 95°C for 10 min followed by 45 cycles of amplification (95°C for 10 s, 60°C for 15 s, 72°C for 10 s) and finally a melting curve program. Target mRNA expression was quantified relative to the housekeeping gene *Hprt1* using the −2ΔΔCt method [26].

### Western Blot

Total protein was extracted from approximately 100 mg of jejunal tissue after removal of the neuromuscular layer by gentle dissection, using T-PER extraction buffer (Thermo Scientific, Waltham, USA). Protein concentration was determined by the Pierce bicinchoninic acid assay (Thermo Scientific) according to the manufacturer’s specifications. Equal amounts of protein were loaded on a 4–12% SDS-PAGE gel and transferred onto a PVDF membrane. The membrane was incubated overnight at 4°C with rabbit anti-claudin 1 (CLDN1) (1∶100; Abcam, Cambridge, UK) or anti-claudin 2 (CLDN2) (1∶500; Abcam) or during 1 hour at room temperature with mouse anti-vinculin (1∶10000; Sigma-Aldrich), which served as the loading control. Anti-rabbit or anti-mouse secondary antibody (1∶5000; Thermo Scientific) was applied during 1 hour at room temperature. Bands were quantified by densitometry using ImageJ software (National Institutes of Health; Bethesda, MD, USA http://rsb.info.nih.gov.ij/) and relative expression compared to the control group was calculated.

### Histology

A 1 cm section of mid-jejunum was fixed overnight in paraformaldehyde 3.7% and embedded in paraffin. Sections of 5 µm were cut using a microtome and stained with hematoxylin and eosin (H&E). Polymorphonuclear (PMN) granulocytes were counted in the lamina propria in 4 high power fields (HPF; 0.233 mm^2^) and in 50 ganglia of the myenteric plexus by an experienced pathologist (GDH) who was blinded to the age and the strain of the animals. A ganglion was defined as a collection of at least two adjacent neuronal cell bodies. The average number of PMN cells per HPF and the total number of PMN cells in 50 ganglia was calculated per animal.

Fifty days was selected as the appropriate time point for immunofluorescence of tight junction proteins, based on the *in vitro* permeability data. Slides were deparaffinized and rehydrated followed by an antigen-retrieval procedure (120°C for 10 minutes in sodium citrate buffer pH 6) and blocking of unspecific binding with Protein Blocking Solution (Dako, Glostrup, Denmark). Slides were incubated with antibodies to CLDN1 (1∶200), CLDN2 (1∶200), zonula-occludens 1 (ZO-1) (1∶50; Invitrogen, Gent, Belgium) and occludin (OCLN) (1∶25; Invitrogen) for 1 hour at room temperature. After washing, slides were incubated with the secondary antibody for 30 min at room temperature: Alexa 594 donkey anti-rabbit (CLDN1), Alexa 488 donkey anti-rabbit (CLDN2 and OCLN) and Alexa 594 donkey anti-mouse (ZO-1) (All 1/1000; Invitrogen). Confocal images were obtained with the LSM510 Meta Laser Scanning microscope (Zeiss, Oberkochen, Germany) from the Cell Imaging Core (CIC, KULeuven). The average fluorescence intensity at the apical intercellular junction was measured in 10 non-overlapping HPF (0.094 mm^2^) using Image Pro (WaveMetrics Inc, OR, USA). Average fluorescence intensity was calculated per animal.

Nitrergic neurons were visualized by a Nicotinamide Adenine Dinucleotide Phosphate – diaphorase (NADPH)-diaphorase histochemical staining [27]. We and others previously demonstrated co-localization of neurons positive for NADPH-diaphorase with neurons positive for neuronal nitric oxide synthase [27]–[29]. Briefly, whole mount preparations of the muscularis propria of the jejunum were prepared by gentle dissection followed by a 40 min fixation step in 3.7% paraformaldehyde at room temperature. After a washing and a permeabilization (0.5% Triton X-100 in phosphate-buffered saline) step, the tissues were incubated for 1 hour at 37°C with tetrazolium blue (0.16 mg/mL) and NADPH (0.8 mg/mL) (Sigma-Aldrich, Diegem, Belgium). The number of blue neurons per HPF (0.933 mm^2^) were quantified in 10 non-overlapping fields per slide and expressed as the average number per HPF.

### Muscle Organ Bath

Nitrergic innervation of the longitudinal smooth muscle was characterized as previously reported. [17] Briefly, jejunal muscle strips (length ±15 mm, width 1.5 mm) were prepared and suspended along their longitudinal axis in a conventional organ bath filled with Krebs solution held at 37°C and gassed with carbogen. All experiments were carried out under non-adrenergic non-cholinergic (NANC) conditions by adding atropine (10^−6^ M) and guanethidine (4×10^−6^ M) to the bath. Neuronal responses were elicited by electrical field stimulation (EFS), applied via two parallel platinum electrodes using a Grass S88 stimulator (Grass, Quincy, MA, USA). Frequency spectra (1–8 Hz) were obtained by pulse trains (pulse 0.35 ms, train 10 s, 8 V). The voltage was kept at 8 V by use of a Med Lab Stimu-Splitter II (Med Lab, Loveland, CO, USA). In between each pulse train, a 90 seconds recovery period was included. Contractions or relaxations were measured using an isometric force transducer/amplifier (Harvard Apparatus, South Natick, MA, USA), recorded on a multi-recorder and sampled for digital analysis using the Windaq Data Acquisition system and a DI-2000 PGH card (Dataq Instruments, Akron, OH, USA).

Before each EFS spectrum, the strips were precontracted by addition of serotonin (5-HT, 3×10^−6^ M) to achieve a stable tone. The nitrergic contribution to the total relaxation under NANC conditions was quantified by repeating the spectrum in the presence and absence of the NOS inhibitor Nitro-L-arginine methyl ester (L-NAME; 3×10^−4^ M) which was added 15 minutes before each EFS spectrum. The responses to EFS were calculated as the mean responses during the stimulation period (on-response), expressed as percentage of the pre-contraction by 5-HT and corrected for the cross-sectional area of the muscle strip. The cross-sectional area was determined as mass tissue wet weight (mg)/tissue length (mm) × density (1.05 mg mm^−3^). The nitrergic contribution was calculated as the reduction of EFS-induced muscle relaxation by L-NAME.

### Statistical analysis

Two-way ANOVA with a post-hoc t-test per time point with Bonferroni correction for multiple testing, was used to evaluate the evolution of the variables (TEER, MPO, etc.) over time in the two groups. Differences between two groups were tested by two-tailed unpaired Student’s t-tests or Mann Whitney U tests depending on the distribution of the data, which was checked by a Kolmogorov-Smirnov test. Differences between more than two groups were evaluated by ANOVA with post-hoc t-test and Bonferroni correction for multiple testing or Kruskal Wallis test with Dunn’s multiple comparison test, when appropriate. Correlations were tested by computing Pearson’s or Spearman’s correlation coefficient depending on the distribution of the data. The muscle strip data were analyzed by a repeated-measures two-way ANOVA with post-hoc Bonferroni correction for multiple testing at the different EFS frequencies. Data are presented as mean ± standard error of the mean (SEM) or median (interquartile range (IQR)) depending on the distribution. Data were analyzed using Prism 5.01 (GraphPad Software, San Diego, CA, USA).

## Results

### Early-onset defect in small intestinal barrier function

**Figure 1:**
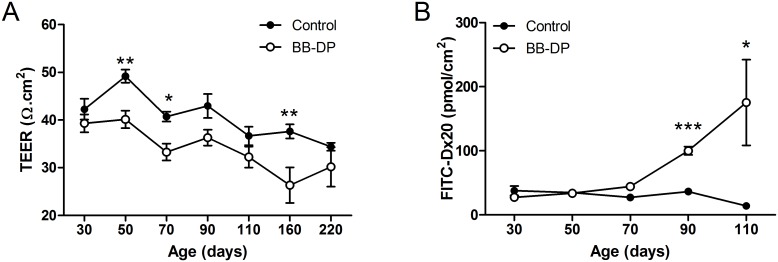
Early increased intestinal permeability. (A) TEER was lower in BB-DP (white circles) rats compared to controls (black circles) starting from 50 days, indicating increased permeability. (B) Increased cumulative passage of FITC-Dx20 at 70, 90 and 110 days, but only the effect at 90 and 110 days remained significant after correction for multiple testing. Data are presented as mean ± SEM. *P<0.05; **P<0.01; ***P<0.001 after correction for multiple testing. BB-DP: normoglycemic Diabetes-Prone BioBreeding; FITC-Dx20: Fluorescein-isothiocyanate labeled dextrans of 20 kiloDalton; TEER: transepithelial-electrical resistance.

BB-DP rats showed signs of impaired intestinal barrier function from an early age, demonstrated by a lower TEER compared to controls starting from 50 days (Fig. 1A). Paracellular passage of FITC-Dx20 was higher in the BB-DP animals at 70 (44.5±5.2 vs. 27.4±4.9 pmol/cm^2^ in BB-DP vs. controls respectively; P<0.05), 90 (100.1±6.5 vs. 36.7±3.1 pmol/cm^2^; P<0.0001) and 110 days (175.5±67.0 vs. 14.2±4.0; P<0.05), but only the difference at 90 and 110 days remained significant after correction for multiple testing (Fig. 1B). These findings were confirmed *in vivo* by a lactulose-mannitol differential urinary excretion test showing an increased urinary LMR at 70 (0.69±0.11 vs. 0.44±0.20; P<0.01) and 90 days (0.39±0.05 vs. 0.28±0.01; P = 0.05), but not at 50 days (0.54±0.05 vs. 0.46±0.03; P = 0.19).

**Figure 2:**
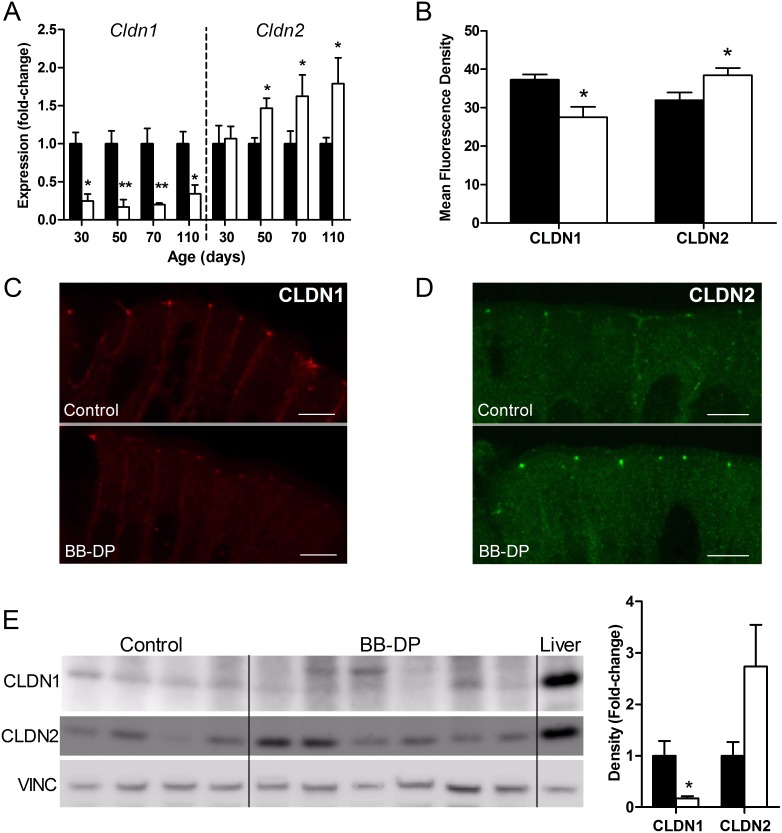
Molecular mechanisms of impaired intestinal barrier function. (A) Lower gene expression of CLDN1 was found at 30, 50, 70 and 110 days and increased expression of CLDN2 at 50, 70 and 110 days in BB-DP rats (white bars) compared to controls (black bars). (B-D) Semi-quantitative immunofluorescence confirmed decreased protein expression of CLDN1 (B, C) and increased expression of CLDN2 (B, D) at 50 days. (scale bar = 10 µm). (E) Western Blot analysis at 50 days shows decreased CLDN1 expression and a tendency towards increased CLDN2 expression (P = 0.07) in BB-DP rats (white bars) compared to controls (black bars). Liver tissue was used as a positive control. Data are presented as mean ± SEM. *P<0.05; **P<0.01. BB-DP: normoglycemic Diabetes-Prone BioBreeding; CLDN1: Claudin 1; CLDN2: Claudin 2; VINC: Vinculin.

To further unravel the molecular mechanism behind the observed intestinal barrier defect, the expression of the intestinal tight junction proteins CLDN1, CLDN2, ZO-1 and OCLN was determined. The gene expression of *Cldn1* was significantly lower in the mucosa of BB-DP animals at all tested time points (Fig. 2A). Expression of *Cldn2*, a pore-forming claudin known to increase permeability [11], [30], was upregulated at 50, 70 and 110 days (Fig. 2A). There were no differences in the expression of *Tjp1*, coding for ZO-1, and *Ocln* at 30, 50, 70 or 110 days (Figure S1A). The gene expression data were confirmed by semi-quantitative immunofluorescence at the tight junction level at the age of 50 days: the mean intensity of CLDN1 was significantly lower and the intensity of CLDN2 was higher in BB-DP rats (Fig. 2B–D). Intensity of ZO-1 and OCLN staining was comparable in both strains (Figure S1B). We did not observe cytoplasmic internalization of tight junction proteins. The lower protein level of CLDN1 and higher expression of CLDN2 were corroborated by western blot analysis, although the increased protein expression of CLDN2 failed to reach full statistical significance (P = 0.07) (Fig. 2E).

### Mucosal and neuromuscular inflammation

**Figure 3:**
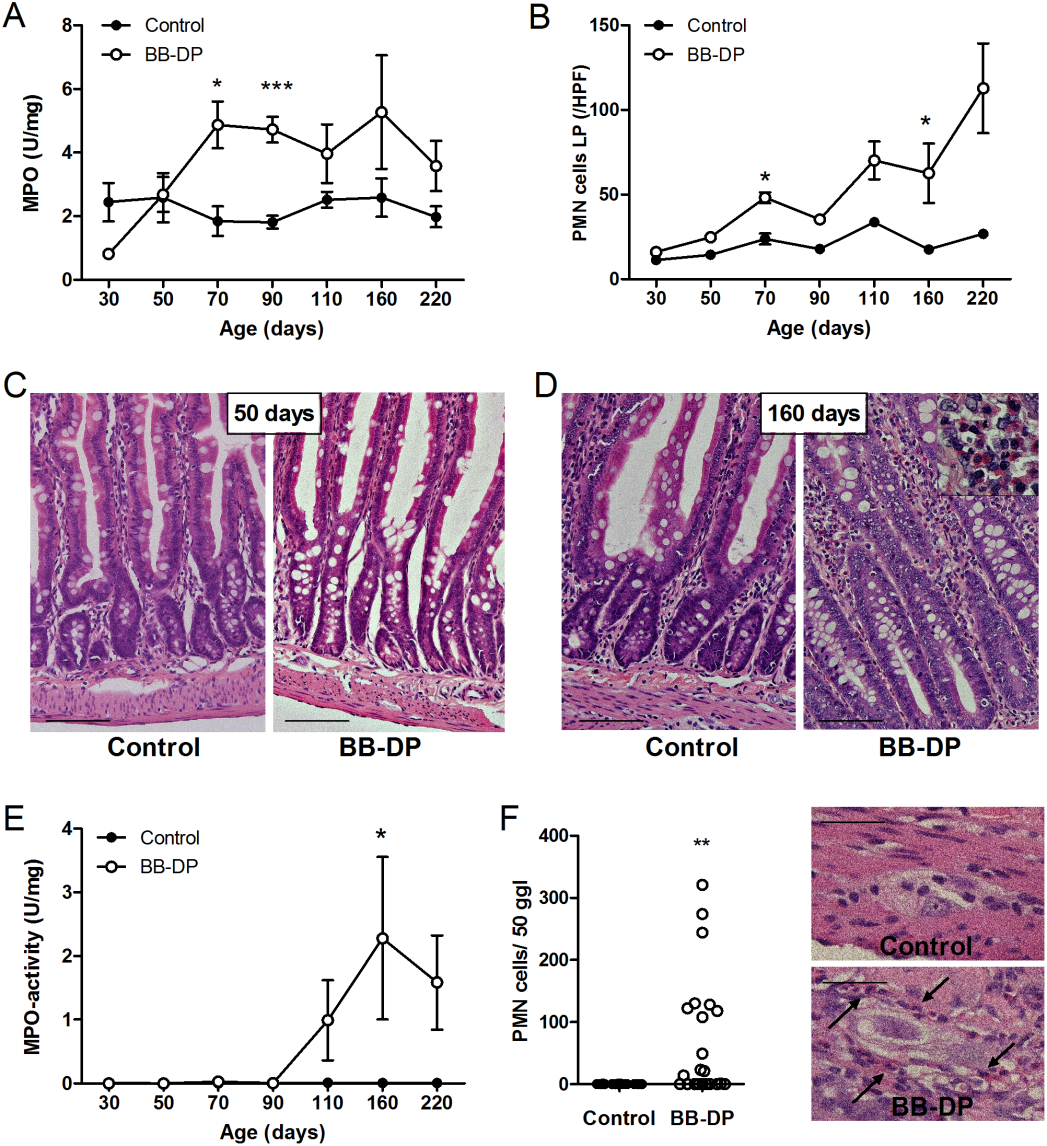
Intestinal inflammation. (A) From 70 days of age, the mucosal MPO-activity was increased in the BB-DP rats (white circles) vs. controls (black circles), however statistical significance was lost in animals older than 110 days. (B) The number of PMN cells per HPF (0.233 mm^2^) in the lamina propria was elevated starting from 70 days. Jejunal histology (H&E staining) at 50 (C) and 160 (D) days (scale bar = 100 µm). (E) In rats older than 110 days, MPO-activity was also elevated in the muscularis propria, but reached significance only at the age of 160 days. (F) Quantification of the number of intra-ganglionic PMN cells in the myenteric plexus in BB-DP (white circles) vs. control (black circles) rats. Intra- and periganglionic PMN cells in BB-DP rats are indicated by black arrows (Scale bar = 25 µm). Data are presented as mean ± SEM. *P<0.05; **P<0.01; ***P<0.001. BB-DP: normoglycemic Diabetes-Prone BioBreeding; HPF: High-Power Field; MPO: Myeloperoxidase; PMN: Polymorphonuclear.

In young rats of 30 and 50 days old, no difference in mucosal MPO activity was detected between the two strains. However, starting from 70 days, MPO activity increased in the BB-DP rats, which was statistically significant at 70 and 90 days after correction for multiple testing (Fig. 3A). In older animals, the MPO activity was numerically higher in BB-DP rats but with a higher variability compared to young animals. As a histological confirmation, the number of PMN cells in the lamina propria was elevated in the BB-DP strain from the age of 70 days and increased further with time (Fig. 3B–D). After correction for multiple testing, this increase remained significant at 70 and 160 days. Starting at 110 days, inflammation also appeared at the level of the muscularis propria, demonstrated by elevated MPO-activity and a myenteric ganglionitis (Fig. 3E–F). Together, these data suggest a progressive inflammatory reaction initiated at the mucosal level and eventually reaching the myenteric ganglia. The inflammation was not evident macroscopically: no erosions, ulcers, perforations or fistulas were present at any time point. Moreover, the weight of the animals was comparable in both groups (462±7 vs. 447±14 g in BB-DP vs. control animals at 220 days; P = 0.32).

Grouping the histological data of 110 to 220 days, i.e. the time points at which transmural inflammation was observed, it became clear that only 12 out of the 22 BB-DP animals evolved to a myenteric ganglionitis (Fig. 3F). Based on this observation, we subclassified these older BB-DP rats into two groups with and without myenteric ganglionitis, based on the presence of PMN cells in the myenteric ganglia (more than the P90 (90^th^ percentile) of PMN cells/50 ganglia in controls = 1). The subgroup of BB-DP rats with myenteric ganglionitis was characterized by more PMN cells in the lamina propria, higher mucosal and muscular MPO values and lower TEER compared to the control group, but also compared to the non-ganglionitis subgroup of the BB-DP rats, which behaved similarly to the control group (Table 1). Furthermore, a moderate inverse correlation between the TEER and mucosal MPO-values (r = –0.76; P<0.001) and PMN cell counts in the lamina propria (r = –0.69; P<0.001) was found in these older BB-DP animals. In contrast, in younger animals (50–90 days), no significant correlations between TEER and inflammatory markers were present (data not shown).

**Table 1:** Inflammatory markers, intestinal barrier function and nitrergic neurons in BB rats older than 110 days.

	Control	BB-DP withoutmyenteric ganglionitis	BB-DP withmyenteric ganglionitis
PMN cells MyentericGanglia (/50 ggl)	0 (0–0)*** (n = 25)	0 (0–0)*** (n = 10)	118 (36–202) (n = 12)
MPO activity MuscularisPropria (U/mg)	0.006 (0–0.014)*** (n = 24)	0.006 (0.002–0.013)** (n = 8)	1.708 (0.169–6.217) (n = 10)
PMN cells LaminaPropria (/HPF)	24.7±2.0*** (n = 19)	41.6±4.3*** (n = 8)	122.5±17 (n = 12)
MPO activity Mucosa(U/mg)	2.40±0.27** (n = 16)	2.89±0.80 (n = 7)	4.96±1.03 (n = 9)
TEER (Ω.cm^2^)	36.2±0.8*** (n = 25)	34.9±1.0*** (n = 10)	21.8±2.5 (n = 7)
FITC-Dx20 flux(110 days) (pmol/cm^2^)	14.2±4.0** (n = 6)	19.1±4.7** (n = 5)	300.6±85.4 (n = 5)
NADPH-diaphorasepositive neurons (/HPF)	40.9±2.9** (n = 21)	36.8±2.6* (n = 8)	16.9±3.0 (n = 6)

The BB-DP strain was divided in a ganglionitis and non-ganglionitis group based on the presence of PMN cells in the myenteric ganglia. Data are expressed as median (IQR) or mean ± SEM depending on the distribution of the data.

*P<0.05;

**P<0.01;

***P<0.001; all vs. BB-DP with myenteric ganglionitis.

BB-DP: normoglycemic Diabetes-prone BioBreeding rat; HPF: High-Power Field; PMN: Polymorphonuclear; MPO: Myeloperoxidase; TEER: Transepithelial Electrical Resistance.

Several cytokines, including interferon (IFN) γ, tumor necrosis factor (TNF) α, interleukin (IL) 1β and IL13, have been implicated in the induction of increased permeability [10], [11]. In young (50–70 days) BB-DP rats, the mRNA expression of these cytokines was lower (IFNγ, IL13) or unaltered (TNFα and IL1β) in comparison to control animals (Table 2), arguing against an important activation of the adaptive immune system in the early phase of impaired mucosal integrity in the BB-DP rat. In contrast, IL13 was significantly increased and there was a tendency towards increased expression of IL1β (P = 0.055) in the ganglionitis subgroup in the older (>110 days) BB-DP animals (Table 3). The expression of the Th1 cytokine IFNγ and the pro-inflammatory TNFα was similar across groups. Furthermore, in BB-DP animals older than 110 days, a higher expression of *Nos2*, coding for the inducible isoform of nitric oxide synthase (iNOS) which is mainly produced by macrophages [31], was detected in the mucosa (Table 3).

**Table 2:** Gene expression analysis of inflammatory markers in BB rats of 50–70 days expressed as fold-changes compared to the control group.

	Age	Control	BB-DP	P-value
IFNγ	50	0.94 (0.69–1.58)	0.27 (0.05–0.57)	**0.005**
	70	1.03 (0.85–1.40)	0.72 (0.37–1.47)	0.45
IL13	50	1.08 (0.84–1.42)	0.30 (0.08–0.58)	**0.04**
	70	1.18 (0.75–1.90)	0.53 (0.07–0.64)	**0.04**
IL1β	50	0.94 (0.67–1.67)	0.59 (0.36–1.90)	0.29
	70	0.97 (0.81–1.53)	1.13 (0.74–1.33)	0.62
iNOS	50	1.06 (0.66–1.51)	1.16 (0.82–2.66)	0.51
	70	0.99 (0.80–1.16)	0.51 (0.24–1.02)	0.16
TNFα	50	1.01 (0.87–1.56)	0.59 (0.26–2.01)	0.23
	70	1.15 (0.57–1.79)	0.73 (0.54–0.88)	0.21

Data are expressed as median (interquartile range). (n = 8 animals per group at 50 days and n = 7 animals per group at 70 days).

BB-DP: normoglycemic Diabetes-Prone BioBreeding Rat; IFN: Interferon; IL: Interleukin; iNOS: inducible isoform of Nitric Oxide Synthase; TNF: Tumor-Necrosis Factor.

**Table 3:** Gene expression analysis of inflammatory markers and permeability-related genes in BB rats older than 110 days, expressed as fold-changes compared to the control group.

	Control (n = 23)	BB-DP without myenteric ganglionitis (n = 10)	BB-DP with myenteric ganglionitis (n = 9)
IFNγ	1.07 (0.54–1.89)	0.87 (0.20–1.52)	0.90 (0.44–2.45)
IL13	1.16 (0.54–1.76)	1.89 (1.06–2.62)	12.39 (8.71–103.1)† ^,^ ‡
IL1β	0.93 (0.64–1.30)	0.76 (0.72–1.40)	2.09 (1.12–2.62)
iNOS	1.17 (0.63–1.56)	3.71 (0.63–11.2)*	7.47 (4.55–18.9)†
TNFα	1.00 (0.49–1.70)	0.97 (0.67–1.20)	1.50 (0.90–2.14)

Data are expressed as median (interquartile range).

*P<0.05 vs. controls;

† P<0.001 vs. controls;

‡ P<0.05 vs. BB-DP without myenteric ganglionitis. BB-DP: normoglycemic Diabetes-Prone BioBreeding Rat; IFN: Interferon; IL: Interleukin; iNOS: inducible isoform of Nitric Oxide Synthase; TNF: Tumor-Necrosis Factor.

### Loss of nitrergic muscle relaxation

**Figure 4:**
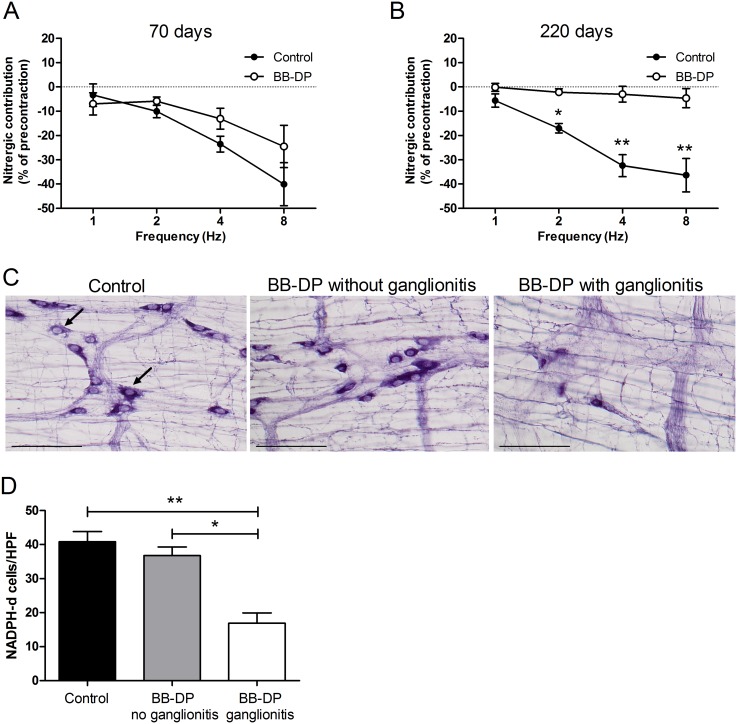
Loss of nitrergic neurons and nitrergic inhibitory motor neuron function. (A) The nitrergic component of EFS-induced relaxation under NANC conditions of longitudinal muscle strips was similar in young (70 days) BB-DP (white circles) and control rats (black circles). (B) However, at 220 days, the nitrergic neuronal function was profoundly reduced in BB-DP-rats. (C) Representative pictures of NADPH-d positive neurons (examples are indicated by the arrows in the control panel) in three animals of the control strain and BB-DP strain with and without ganglionitis of 160 days are shown. (Scale bar = 100 µm) (D) The number of NADPH-d positive neurons, was lower in the subgroup of the BB-DP rats with myenteric ganglionitis (white bar) in comparison to controls (black bar) and BB-DP rats without myenteric ganglionitis (gray bar) of 110 to 220 days old. Data are presented as mean ± SEM. *P<0.05; **P<0.01. BB-DP: normoglycemic Diabetes-Prone BioBreeding; EFS: Electrical Field Stimulation; NADPH-d: Nicotinamide Adenine Dinucleotide Phosphate diaphorase; NANC: non-adrenergic non-cholinergic.

At the age of 70 days, the nitrergic contribution to the EFS-induced muscle relaxation was similar in both groups (Fig. 4A). Likewise, the density of nitrergic neurons, identified by a positive NADPH-diaphorase staining, was unaltered in young BB-DP rats of 70 days (53±3 vs. 51±7/HPF in BB-DP vs. controls; P = 0.70). In contrast, the nitrergic component of the relaxation under NANC conditions was significantly reduced in BB-DP animals of 220 days (Fig. 4B), indicating a loss of nitrergic inhibitory motor neuron function in older BB-DP rats. Of the 220-day old animals included in the muscle strip experiments, one rat was free from myenteric ganglionitis and also was the only one with preserved nitrergic inhibition. The nitrergic contribution at 8 Hz was 36.4±6.9% (range 16.0–69.9%) vs. 0.9±1.5% (range 0–3.9%) in controls and BB-DP animals with myenteric ganglionitis respectively. The one BB-DP animal without myenteric ganglionitis had a nitrergic contribution of 19.5% of the precontraction, which is far out of the range of the other BB-DP animals and comparable to the control group.

Moreover, the number of nitrergic neurons tended to be lower at 110 (29±4 vs. 38±1; P = 0.07) and was lower at 160 (26±6 vs. 48±3; P = 0.02) and 220 days (30±5 vs. 44±8; P = 0.03) (Fig. 4C), but these differences did not persist after correction for multiple testing. However, grouping the BB-DP animals older than 110 days in a non-ganglionitis vs. ganglionitis phenotype as described above, a significant decrease in NADPH-diaphorase positive neurons was observed in the BB-DP rats with myenteric ganglionitis (Table 1 and Fig. 4C). The number of nitrergic neurons in the subgroup of the BB-DP animals without myenteric ganglionitis was comparable to the control strain.

## Discussion

In the current study we described the complex chain of events leading from early impaired mucosal integrity to myenteric ganglionitis and dysmotility in a spontaneous rat model of leaky gut. We demonstrated the presence of intestinal hyperpermeability prior to the development of mucosal immune activation in BB-DP rats, suggesting a disease-initiating role. At later time points, half of the animals developed a transmural inflammatory reaction with myenteric ganglionitis. Finally, in the older rats with intra-ganglionic inflammation, a loss of nitrergic neurons and function was observed. Our findings suggest that impaired mucosal integrity can give rise to a transmurally progressing inflammation resulting in disturbed motility, providing an insightful model for human FGID.

Increased intestinal permeability and low-grade inflammatory alterations in the gastrointestinal tract have been suggested to contribute to the pathogenesis of FGID [3]–[6]. A cascade in which intestinal permeability represents the first hit, leading to immune activation and subsequent neuromuscular alterations, is often proposed in the literature [4], [5]. However, because of the lack of drugs which can restore the leaky barrier and the fact that patients will only present themselves at the time of symptoms, it is challenging to separate cause from effect in humans [11]. In the current study in BB-rats, we found an impaired barrier function in young rats of 50 days, a time point at which no immune cell infiltration or increased MPO-activity was observed. Furthermore, the expression of TNFα, IFNγ, IL1β and IL13 was not increased in these young rats. This is of particular importance since it is well established that these cytokines can impair intestinal barrier function [32]–[36]. We speculate that the initial defect in the intestinal barrier leads to the subepithelial penetration of unprocessed antigens, inciting an inflammatory reaction, which in turn maintains the permeability defect at the later time points. These events may lead to a vicious cycle as suggested by the correlation between the permeability and inflammatory parameters in the older animals and by the fact that the permeability parameters in the older animals without ganglionitis were comparable to controls. In order to definitely confirm this hypothesized biphasic permeability defect, experiments using anti-inflammatory treatments are warranted.

The barrier function of the intestinal mucosa is regulated primarily by the apical junction complex, of which the tight junction is the most critical component. The tight junction consists of transmembrane proteins, of which the claudins and OCLN are the best characterized, and linkage proteins such as ZO-1 which connect the transmembrane proteins to the cytoskeleton. In the BB rat model, the expression of CLDN1, one of the main sealing claudins of the intestine, was lower compared to controls at all investigated time points, also at 30 days when the permeability parameters were still similar in both strains. The reason for lower CLDN1 expression is unclear, but it may represent a genetic susceptibility. Our data are in partial agreement with Visser *et al.* who reported decreased CLDN1 expression in the ileum of 50 to 70 day old BB-DP rats, but not at 30–50 days [16]. It is unclear whether regional variation, i.e. jejunum vs. ileum, or a different substrain may explain the difference in time of onset. A lower expression of CLDN1 has also been reported in one study in patients with diarrhea-predominant IBS [37], although not confirmed by others [38]. Only from the age of 50 days, intestinal permeability increased in BB-DP animals, coinciding with an enhanced expression of CLDN2, a pore-forming claudin that enhances permeability [39]. Combined, these data suggest that in our model lower expression of CLDN1 is insufficient to induce increased permeability and only the combination with increased CLDN2, of which the trigger has not been identified yet, leads to the barrier defect. Increased expression of CLDN2 has also been reported in intestinal biopsies of patients with IBD [40], [41] and IBS [38]. However, the exact mechanisms by which the overexpression of CLDN2 can contribute to disease pathogenesis are still elusive since only small, supposedly non-immunogenic, solutes can permeate through CLDN2 pores. Intriguingly, the mucosal-to-serosal flux of the 20 kDa dextran was unchanged at 50 days, while a clear decrease in TEER was present. At the later time points, a progressive rise in dextran passage was observed, in contrast to a stable difference in TEER. At least two different routes of intestinal paracellular flux have been reported in literature: a large-capacity pathway for small solutes (less than 4 Å) and water, the ‘pore pathway’, and a small-capacity pathway for larger molecules, the ‘leak pathway’ [30]. The mucosal-to-serosal flux of macromolecules is regulated by the leak pathway, while TEER reflects a combination of both pore and leak pathway. Although cross-talk exists to some extent, both pathways are regulated separately. Our data suggest that the pore pathway is affected first in BB-DP animals, while the defect in the leak pathway only follows at later time points. However, we cannot exclude that the size of the dextran used in our experiments prevented us from detecting early differences in the leak pathway. The combination of different sizes of tracers could help to answer this question, which, however, was beyond the scope of the current study.

The observed alterations in permeability and the fact that inflammation progresses over time from the mucosa to the muscle layer, point towards the involvement of a luminal factor. Previous studies have supported this hypothesis, showing a normalization of the barrier function and tight junction composition in BB-rats on a hydrolyzed casein diet [16], [21], [42]. However, the effects of dietary modification on mucosal inflammation, myenteric ganglionitis and intestinal motility are unknown and subject for further research. In keeping with the hypothesis of a luminal contributing factor in human FGID, fecal supernatants of patients with diarrhea predominant IBS led to increased permeability and visceral hypersensitivity when injected intracolonically in mice [43]. Some groups have advocated a role for gluten as a luminal contributor in IBS [44], [45], although this was challenged by others [46]. In a randomized controlled trial in non-celiac IBS patients, a gluten-containing diet was associated with significantly higher small intestinal permeability and altered expression of tight junction proteins in comparison to a gluten-free diet, especially in individuals who were HLA-DQ2/8 positive [45]. Finally, in *ob/ob* mice, increased intestinal permeability was reported which was dependent on the gut microbiota and could be reversed by treatment with prebiotics [47]. Together, the available data suggest a luminal, possibly food-related, factor in intestinal hyperpermeability, both in IBS patients and the BB-rat. Further studies to identify the triggering food constituents or other players like proteases and bacteria are awaited.

The intestinal immune activation in the BB-rat was characterized by infiltration of PMN cells and increased MPO-activity. The full characterization of the inflammatory infiltrate was beyond the scope of the current study. Histologically, PMN cells in our study may encompass neutrophils, eosinophils and mast cells. However, the combination with increased MPO activity suggests a predominantly neutrophilic infiltrate. Although most studies have focused on the increased presence of eosinophils, mast cells and lymphocytes in FGID [4], neutrophils have been reported as well [48], [49]. We acknowledge that also monocytes and classically activated macrophages are known to express MPO [50], but the absence of significant iNOS upregulation in young rats arguments against macrophages as an important player in the early inflammatory phase, although they do seem to participate in the older rats as demonstrated by iNOS overexpression after 110 days of age. Although at this point we cannot exclude or confirm the presence of mast cells and eosinophils in our model, since they are not easily discernible on H&E staining in rodents, we have observed only few lymphocytes. The absence of a major lymphocytic infiltration is probably related to the lymphopenic nature of the model due to the mutation of the *Gimap5* or *lyp* gene [51].

An intriguing observation was that only around 50% of the animals developed a transmural inflammatory reaction and loss of nitrergic neurons, even though they were litter mates, sharing the same environment, chow and genetics. We were not able to identify subgroups in the younger age group, suggesting that the early hyperpermeability is a general, strain-specific defect, while transmural inflammation and the secondary permeability defect depend on additional, unknown factors.

Due to the requirement of full-thickness biopsies, the data on neuromuscular inflammation in IBS are limited to two studies by Tornblom et al. The first study demonstrated a low-grade lymphocytic myenteric ganglionitis in 9 out of 10 patients with severe IBS [7]. More recently, the same group published a follow-up study showing a lymphocytic infiltrate in 48/65 patients with enteric dysmotility, a newly described FGID entity characterized by severe abdominal symptoms and dysmotility on small bowel manometry [52]. It is unknown what proportion of patients with functional dyspepsia and IBS actually show enteric dysmotility and myenteric plexus abnormalities.

Possible mechanisms of symptom generation in FGID include direct activation of sensory neurons by immune mediators [53], but also disturbed motility linked to alterations in the enteric nervous system (ENS) [6], [7], [9]. Research in animal models of post-inflammatory dysmotility indicated a selective loss of inhibitory innervation [54]–[56]. In keeping with this mechanism, we previously reported impaired gastric accommodation in post-infectious functional dyspepsia patients which was linked to an impaired nitrergic relaxation of the fundus [8]. In the current study we confirmed an important reduction of nitrergic neurons in the jejunal myenteric plexus of older BB-rats with myenteric ganglionitis. This finding was confirmed functionally by a decreased nitrergic contribution to the EFS-induced relaxation of the longitudinal smooth muscle under NANC conditions. Recently, we have reported impaired gastric accommodation related to impaired nitrergic inhibition in the BB-DP rat [57]. Additional studies investigating intestinal transit and visceral hypersensitivity are necessary to definitely confirm the BB-DP rat as a model for FGID. Also, it will be worthwhile to investigate whether sex-related differences and psychopathology traits such as anxiety, psychological stress and depression, similar to human FGID, can also be found in this rat model.

A potential weakness of our study is the fact that only jejunum was studied, while most symptoms of IBS are thought to originate from the lower gastrointestinal tract. However, also in IBS patients, abnormalities in permeability and inflammation are present in the proximal small intestine [7], [38]. Moreover, we reported altered permeability and immune activation in the duodenum of patients with functional dyspepsia [5]. Nevertheless, involvement of other segments of the gastrointestinal tract like the colon or the stomach needs to be studied in detail in follow-up studies in the BB-rat.

In conclusion, we propose the BB-rat as a spontaneous animal model to study the pathogenesis of FGID. In the current study we describe the sequence of early impaired mucosal integrity leading to a progressive, transmural inflammatory reaction, ultimately resulting in a myenteric ganglionitis with concomitant loss of nitrergic neurons and disturbed motility in the jejunum. These findings suggest an early pathogenic role for the impaired barrier function in the BB-rat model. The availability of a spontaneous model for FGID will facilitate pre-clinical development of therapies in these challenging disorders.

## Supporting Information

Figure S1 **Molecular mechanisms of impaired barrier function.** (A) Gene expression of *Tjp1*, coding for ZO-1, and *Ocln*, coding for occludin, was similar in BB-DP rats (white bars) compared to controls (black bars). at 30, 50, 70 and 110 days. (B) Semi-quantitative immunofluorescence demonstrated unaltered protein expression for ZO-1 and occludin in both groups at 50 days of age. BB-DP: normoglycemic Diabetes-Prone BioBreeding.
(TIF)

Table S1 **Primer sequences for RT-PCR.**
(DOCX)
