# Supplementary figures and images for: From Intestinal Permeability to Dysmotility: The Biobreeding Rat as a Model for Functional Gastrointestinal Disorders
Source: PLoS One. 2014 Oct 29;9(10):e111132. doi: 10.1371/journal.pone.0111132 (PMC4212994; doi:10.1371/journal.pone.0111132)

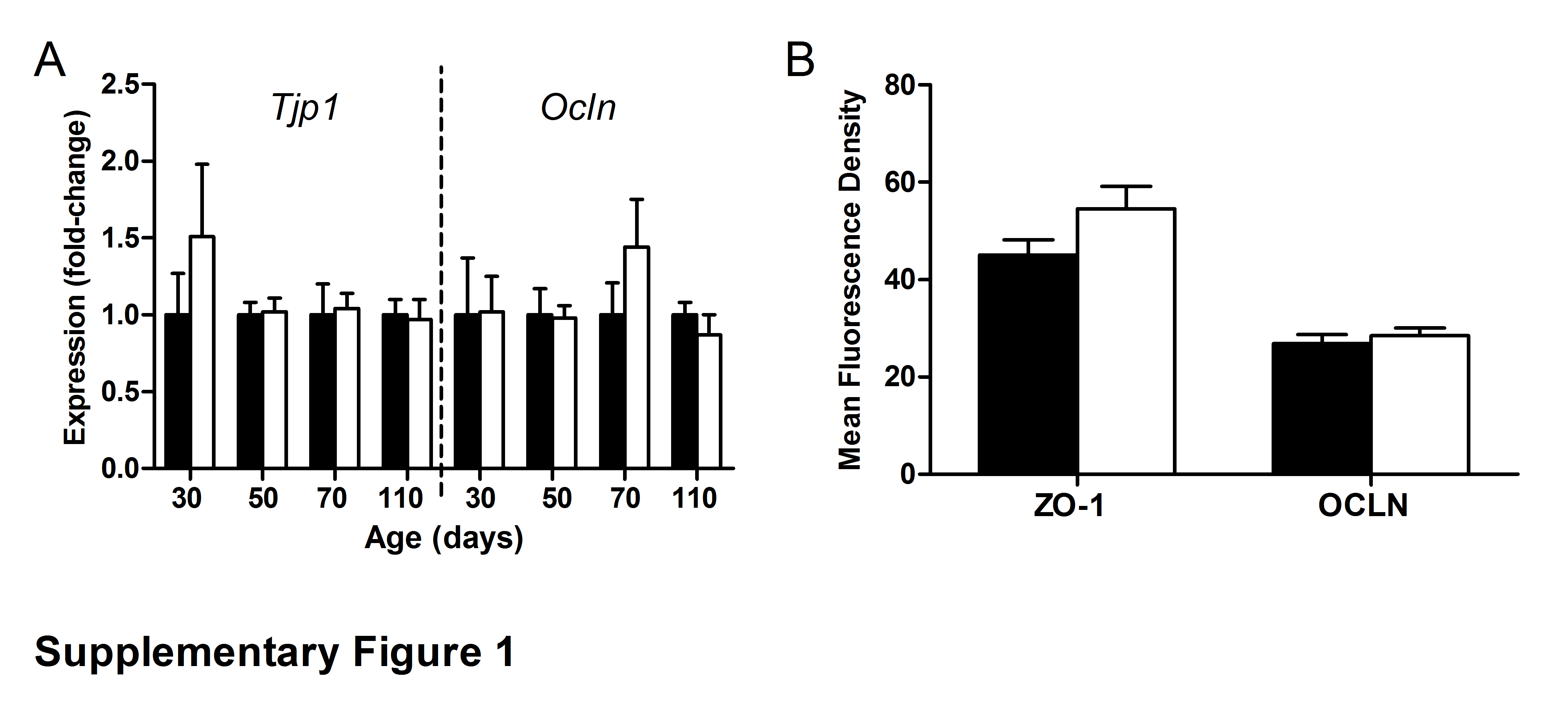

Supplement: Figure S1 — Molecular mechanisms of impaired barrier function. (A) Gene expression of Tjp1, coding for ZO-1, and Ocln, coding for occludin, was similar in BB-DP rats (white bars) compared to controls (black bars). at 30, 50, 70 and 110 days. (B) Semi-quantitative immunofluorescence demonstrated unaltered protein expression for ZO-1 and occludin in both groups at 50 days of age. BB-DP: normoglycemic Diabetes-Prone BioBreeding. (TIF) [file pone.0111132.s001.tif]
