# Supplementary material for: From Intestinal Permeability to Dysmotility: The Biobreeding Rat as a Model for Functional Gastrointestinal Disorders
Source: PLoS One. 2014 Oct 29;9(10):e111132. doi: 10.1371/journal.pone.0111132 (PMC4212994; doi:10.1371/journal.pone.0111132)
Supplement: Table S1 — Primer sequences for RT-PCR. (DOCX) [file pone.0111132.s002.docx]

**SUPPLEMENTARY TABLES**

**Table S1**

| Gene | Sense primer | Antisense primer |
| --- | --- | --- |
| *Hprt1* | GCGAAAGTGGAAAAGCCAAGT | GCCACATCAACAGGACTCTTGTAG |
| *Cldn1* | ATTGGCATGAAGTGCATGAG | CCACTAATGTCGCCAGACCT |
| *Cldn2* | GGCTATTAGGCACATCGAT | TGGCACCAACATAAGAACTT |
| *Ifng* | TATCTGGAGGAACTGGCAAAAG | TGCGATTCGATGACACTTATGT |
| *Il1b* | CGTGGAGCTTCCAGGATGAG | CGTCATCATCCCACGAGTCA |
| *Il13* | CCAGTGCGGAGATCCACATC | GGTCCACGCTCCATACCATG |
| *Nos2* | ACCCAAGGTCTACGTTCAAGACA | CACATCCCGAGCCATGC |
| *Ocln* | ATGTCTGTGAGGCCTTTTGA | TACATGTCATTGCTTGGTGC |
| *Tjp1* | CCCTCTGATCATTCCACACA | TTTAGACATGCGCTCTTCCT |
| *Tnfa* | GATCGGTCCCAACAAGGAGG | GCTTGGTGGTTTGCTACGAC |

Primer sequences for RT-PCR
